# Phloroglucinol-Mediated Hsp70 Production in Crustaceans: Protection against *Vibrio parahaemolyticus* in *Artemia franciscana* and *Macrobrachium rosenbergii*

**DOI:** 10.3389/fimmu.2018.01091

**Published:** 2018-05-22

**Authors:** Vikash Kumar, Kartik Baruah, Dung Viet Nguyen, Guy Smagghe, Els Vossen, Peter Bossier

**Affiliations:** ^1^Laboratory of Aquaculture & Artemia Reference Center, Department of Animal Sciences and Aquatic Ecology, Faculty of Bioscience Engineering, Ghent University, Ghent, Belgium; ^2^ICAR-Central Inland Fisheries Research Institute (CIFRI), Barrackpore, India; ^3^Department of Crop Protection, Faculty of Bioscience Engineering, Ghent University, Ghent, Belgium; ^4^Laboratory of Animal Nutrition and Animal Product Quality, Department of Animal Sciences and Aquatic Ecology, Faculty of Bioscience Engineering, Ghent University, Ghent, Belgium

**Keywords:** gnotobiotic *Artemia*, *Vibrio parahaemolyticus*, phloroglucinol, heat shock protein 70, *Macrobrachium rosenbergii*

## Abstract

The halophilic aquatic bacterium, *Vibrio parahaemolyticus*, is an important aquatic pathogen, also capable of causing acute hepatopancreatic necrosis disease (AHPND) in shrimp resulting in significant economic losses. Therefore, there is an urgent need to develop anti-infective strategies to control AHPND. The gnotobiotic *Artemia* model is used to establish whether a phenolic compound phloroglucinol is effective against the AHPND strain *V. parahaemolyticus* MO904. We found that pretreatment with phloroglucinol, at an optimum concentration (30 µM), protects axenic brine shrimp larvae against *V. parahaemolyticus* infection and induced heat shock protein 70 (Hsp70) production (twofolds or more) as compared with the control. We further demonstrated that the *Vibrio*-protective effect of phloroglucinol was caused by its prooxidant effect and is linked to the induction of Hsp70. In addition, RNA interference confirms that phloroglucinol-induced Hsp70 mediates the survival of brine shrimp larvae against *V. parahaemolyticus* infection. The study was validated in xenic *Artemia* model and in a *Macrobrachium rosenbergii* system. Pretreatment of xenic brine shrimp larvae (30 µM) and *Macrobrachium* larvae (5 µM) with phloroglucinol increases the survival of xenic brine shrimp and *Macrobrachium* larvae against subsequent *V. parahaemolyticus* challenge. Taken together, our study provides substantial evidence that the prooxidant activity of phloroglucinol induces Hsp70 production protecting brine shrimp, *A. franciscana*, and freshwater shrimp, *M. rosenbergii*, against the AHPND *V. parahaemolyticus* strain MO904. Probably, phloroglucinol treatment might become part of a holistic strategy to control AHPND in shrimp.

## Introduction

The acute hepatopancreatic necrosis disease (AHPND), originally known as early mortality syndrome, is a newly emerging shrimp disease causing havoc in the shrimp industry (1). The disease is caused by a specific virulent strain of Gram-negative marine bacterium, *Vibrio parahaemolyticus*, ubiquitous in estuarine and coastal waters (2). Since the AHPND outbreak first appeared in China in 2009, it has spread to Vietnam (2010), Malaysia (2011), Thailand (2012), Mexico (2013), Philippines (2015), and South America (2016) (3, 4). The shrimp production in AHPND-affected regions has dropped to ~60%, and the disease has caused global loss of $1 billion per year to the shrimp farming industry (5, 6). The *V. parahaemolyticus* becomes virulent VP_AHPND_ by acquiring a 63- to 70-kb plasmid (pVA1) encoding the binary toxin PirA^Vp^/PirB^Vp^, which consist of two subunits PirA and PirB, and is homologous to the *Photorhabdus luminescens* insect-related (Pir) toxins PirA/PirB (7–9). Therefore, to enable the prevention of *V. parahaemolyticus*, an approach that focuses on understanding the host immune system and develops effective anti-infective strategies, in general, will have the highest chance of decreasing the risk of pathogenic *V. parahaemolyticus* infection in shrimps.

The conventional approach so far applied in the mitigation or cure of *V. parahaemolyticus* such as antibiotics and disinfectants had limited success (10). In addition, their usage in the food-producing sector is under severe scientific and public scrutiny due to the development of multiple resistances (11). Because of such concerns, there is an urgent need for the development of polyphenol plant-derived compounds or natural products that protect the shrimp and enhance the immune reactivity to *V. parahaemolyticus*. However, the critical problem in studying the anti-pathogenic effects of a polyphenol plant-based compound *in vivo* is the difficulty to either eliminate or extricate the effect of the microbial communities that occur naturally in the system (12). In addition, in germ-associated conditions, the compound of interest is either metabolized by microbial communities or influences the physiology of host-associated microbes, thereby making it difficult to understand the host response toward tested compound (13). Consequently, the selection of appropriate animal model system that permits better delineation of the biological effects of polyphenol plant-based compounds is paramount, for instance, on the induction of resistance to disease.

The brine shrimp (*Artemia franciscana*), an aquatic invertebrate that can be reared under gnotobiotic conditions (allowing full control over the host-associated microbial communities), is characteristically small, highly osmotolerant, branchiopod crustacean reported from a variety of harsh environment worldwide (14). Apart from its unusual life history, relatively low space and cost requirement for culture, rapid generation cycle (cyst to adult in 20–30 days), well-characterized developmental stages, the ability to form cyst that can be stored and used, and established molecular techniques like RNA interference (RNAi) make *A. franciscana* an exceptional experimental system to study the biological activity of polyphenol plant-based compounds as it allows to distinguish the direct effect on the host (by pre-exposing axenic brine shrimp larvae to the compound for a certain duration) from indirect effects (15–18).

Given the potential of polyphenol plant-based compound, phloroglucinol, as an anti-infective agent, understanding the underlying mechanism of action of this compound is considered vital. In this study, using a highly controlled gnotobiotic brine shrimp model system, we aimed to investigate whether phloroglucinol potentiates the generation of prooxidant activity and heat shock protein 70 (Hsp70) production *in vivo* and whether this putative effect could contribute to the induction of protective responses in *Artemia* against pathogenic *V. parahaemolyticus* MO904 strain. Furthermore, we also unraveled the mechanism behind the possible Hsp70-inducing effect of phloroglucinol. The results obtained in the gnotobiotic brine shrimp model system were validated in a xenic brine shrimp and *Macrobrachium* larvae, and we present novel finding which demonstrates that phloroglucinol is indeed a potent *in vivo* enhancer of Hsp70 and this effect mediates the induction of resistance in xenic brine shrimp and *Macrobrachium* larvae against *V. parahaemolyticus*.

## Materials and Methods

### Bacterial Strains for *In Vivo* Challenge Tests

In total, seven bacterial strains were used in the study: *V. parahaemolyticus* (AHPND strains) KM, MO605, RY, MO903, MO904, non-AHPND strain CAIM170, and *Aeromonas hydrophila* LVS3. LVS3 (autoclaved) were used as feed for brine shrimp larvae and MO904 as a pathogen for the challenge assay. The *V. parahaemolyticus* strains were confirmed for AHPND bacteria by PCR using AP3 primers (Table 1). Based on the amplification of DNA sequence of *V. parahaemolyticus* strains, it was confirmed as AHPND-causing strain that is not present in non-AHPND *V. parahaemolyticus* (Figure S1 in Supplementary Material) (19, 20). Before the challenge assay, the virulence of *V. parahaemolyticus* AHPND strains (KM, MO605, RY, MO903, and MO904) and non-AHPND strain (CAIM170) was evaluated using axenic *Artemia* as host. The *V. parahaemolyticus* MO904 strain provoking significant mortality in brine shrimp larvae up to 80% after 48 h was selected for the experimental challenge (Figure S2 in Supplementary Material). The MO904 strain used in the experiment was collected from the collection of aquatic important microorganism, CIAD, A.C. Mazatlàn unit of Aquaculture, Mazatlàn, Sinaloa, Mexico, and the isolates have been transferred to the Laboratory of Aquaculture and *Artemia* Reference Center, Ghent University, Belgium, under material transfer agreement in 2013. The stock culture of MO904 was streaked onto Luria Bertani agar plates (Himedia, India) containing 2% NaCl (LB+) to obtain pure colonies. A single colony was inoculated into LB+ broth (Himedia, USA), incubated overnight at 28°C under constant agitation (100/min), and the stocked culture was prepared in 40% glycerol and stored in −80°C. The LVS3 stock culture collected from the Laboratory of Aquaculture and *Artemia* Reference Center, Ghent University, Belgium, was streaked onto Marine agar plates (Difco Laboratories, Detroit, MI, USA). For cultivation, a single colony was inoculated in Marine broth (Difco Laboratories, Detroit, MI, USA).

**Table 1:** AP3 primers used for confirmation of *V. parahaemolyticus* AHPND strains.

AP3	5′–3′	Length	% GC	Tm	Ta	Expected amplicon
Forward	ATGAGTAACAATATAAAACATGAAAC	26	23.08	57.63	53	336 bp
Reverse	GTGGTAATAGATTGTACAGAA	21	33.33	55.46		

### Reagents

Phloroglucinol (≥99%) purchased from Sigma-Aldrich (Diegem, Belgium) was dissolved in sterile distilled water at 0.4 g/l (3.17 mM). Superoxide dismutase (SOD) (2,000–6,000 units/mg protein) and catalase (2,000–5,000 units/mg protein) were also obtained from Sigma-Aldrich. Catalase was dissolved in sterile distilled water at 0.5 g/l as stock solution. All the stock solutions were prepared fresh for each experiment.

### Gnotobiotic Brine Shrimp Rearing System

The gnotobiotic brine shrimp larvae were produced by hatching brine shrimp cysts axenically (germ-free) following decapsulation and hatching procedures as described by Baruah et al. (21). Briefly, 1.5 g of cysts (EG^®^ type, batch 21452, INVE Aquaculture, Dendermonde, Belgium) were hydrated in 89 ml of distilled water for 1 h. Sterile cysts and larvae were obtained *via* decapsulation using 3.3 ml NaOH (32%) and 50 ml NaOCl (50%). During the reaction, 0.2-µm filtered aeration was provided. All manipulation was carried out under a laminar flow hood and all tools were sterilized. The decapsulation was stopped after 2 min by adding 50 ml Na_2_S_2_O_3_ at 10 g/l. The decapsulated cysts were washed with filtered autoclaved seawater (FASW) containing 35 g/l of instant ocean^®^ synthetic sea salt (Aquarium Systems, Sarrebourg, France). The cysts, suspended in 1-l glass bottles containing FASW, were incubated at 28°C for 28 h for hatching with a constant illumination of approximately 27 μE/m^2^ s. The hatched larvae at developmental stage instar II (mouth is opened to ingest particles) were collected, and the axenicity was verified by spread plating (100 ml) as well as by adding (500-µl) hatching water on Marine Agar and Marine Broth, respectively, followed by incubation at 28°C for 5 days (22). Experiments that started with non-axenic larvae were discarded.

### Brine Shrimp Lethality Test and Challenge Assay

In total, four separate challenge tests were performed. The first test determined the dose–response relationship (protective effect) of phloroglucinol. Briefly, hatched brine shrimp larvae (at developmental stage II) were collected and counted volumetrically. Firstly, the sub-samples (1 ml) were tallied, and this number was multiplied by the total measured volume (ml) of hatched larvae. Thereafter, the larvae were transferred to sterile 50-ml falcon tubes containing 30 ml. The axenic larvae were pretreated with increasing concentrations of phloroglucinol (5, 10, 20, 30, 40, 50, and 100 µM) for 2 h at 28°C. They were repeatedly rinsed with FASW to wash away the compound and then allowed to recover for 2 h at 28°C. Following recovery period, a group of 20 larvae were transferred to sterile 40-ml glass tubes containing 10-ml FASW. Afterward, it was verified whether the pretreatment of phloroglucinol can protect the larvae against subsequent challenge with *V. parahaemolyticus* MO904 strain at 10^7^ cells/ml. The survival of *Artemia* was scored 48 h after the addition of the pathogen. The non-pretreated larvae that were either challenged with *V. parahaemolyticus* or not were maintained as negative and positive controls. Each treatment and control were done in quintuplicate.

In the second test, the cytotoxic effect of phloroglucinol was determined in the axenic brine shrimp larvae as previously described by Baruah et al. (13). The axenic larvae were pretreated with an increasing concentration of phloroglucinol as described above in the dose–response study. Subsequently following recovery period, toxicity assay was performed in sterile 40-ml glass tubes with 20 larvae in 10-ml FASW per tube. The toxicity of the compound was determined after 48 h by scoring the number of survivors as previously described (13). Brine shrimp larvae that did not receive the phloroglucinol pretreatment served as control group. Five replicates were maintained for each treatment and control groups.

The third and fourth tests were performed to determine the mode of action of phloroglucinol. The axenic brine shrimp larvae were pretreated with an optimized dose of phloroglucinol (a dose that gave maximum protection to challenged larvae in the dose–response assay), a mixture of the antioxidant enzymes catalase (10 mg/l) and SOD (75 units), or a combination of phloroglucinol and antioxidant enzymes mixture in a similar manner as described above. The larvae in the control group did not undergo any pretreatment. Groups of 20 larvae were counted and distributed in sterile 40-ml glass tubes and then challenged with *V. parahaemolyticus* MO904 strain as described in the dose–response study. The survival of larvae was scored 2 days after the addition of pathogen. Brine shrimp larvae that did not receive phloroglucinol pretreatment either challenged with *V. parahaemolyticus* or not served as negative and positive controls. Each treatment and control were performed in five replicates.

### Protein Extraction and Detection of Hsp70 in Brine Shrimp Larvae

Following pretreatment with phloroglucinol, antioxidant enzymes, and a mixture of antioxidant enzymes and phloroglucinol, live brine shrimp larvae (0.1 g w/v) were collected, rinsed in distilled water, immediately frozen in liquid nitrogen, and stored in −80°C. *Artemia* samples were homogenized in cold buffer K (150 mM sorbitol, 70 mM potassium gluconate, 5 mM MgCl_2_, 5 mM NaH_2_PO_4_, 40 mM HEPES, pH 7.4) (23), supplemented with protease inhibitor cocktail (Sigma-Aldrich^®^, USA). Subsequently, the samples were centrifuged at 2,200 × *g* for 1 min at 4°C, and the supernatant protein concentration was determined by the Bradford method (24) using bovine serum albumin as standard. Supernatant samples were then combined with loading buffer, vortexed, heated for 5 min at 95°C, and electrophoresed in 10% SDS-PAGE gel (BioRad, Belgium), with each lane receiving an equivalent amount of protein. HeLa (heat-shocked) cells (Enzo Life Sciences, USA) (6 µg) were loaded on to one well to serve as a positive control. Gels were then either stained with Coomassie Biosafe (BioRad Laboratories) or transferred to polyvinylidene fluoride membrane (BioRad Immuno-Blot™ PVDF) for antibody probing. Membrane was incubated with a blocking buffer [50 ml of 1× phosphate-buffered saline containing 0.2% (*v/v*) tween 20 and 5% (*w/v*) bovine serum albumin] for 60 min at room temperature and then with mouse monoclonal anti-Hsp70 antibody (3 A3) (Affinity BioReagents Incl., Golden, CO, USA), at the recommended dilution of 1:5,000 (22). Horseradish peroxidase-conjugated donkey anti-mouse IgG was used as secondary antibody at the recommended dilution of 1:2,500 (Affinity BioReagents Incl., Golden, CO, USA). The membrane was then incubated with clarity western ECL substrate (chemiluminescence reagent) (BioRad Laboratories) for 5 min, and the signals were detected by a ChemiDoc MP imaging system (BioRad, Belgium).

### Lipid Peroxidation

The extent of lipid peroxidation was measured by the methods of Grotto et al. (25), and malondialdehyde (MDA) was determined calorimetrically. In brief, gnotobiotic brine shrimp larvae were pretreated with an optimized dose (30 µM) of phloroglucinol for 2 h at 28°C. They were repeatedly rinsed with FASW to wash away the compound and then allowed to recover for 2 h at 28°C. Following the recovery period, the samples containing 0.1 g (w/v) of live larvae were harvested from treatment and control and immediately frozen in liquid nitrogen and stored at −80°C for further MDA determination. The samples were hydrolyzed in mild alkaline conditions (1.5 M NaOH) at 60°C for 30 min in order to release bound MDA. The mixture was incubated with 6% H_3_PO_4_ and 0.8% thiobarbituric acid at 90°C for 45 min. After cooling, 10% SDS was added and the chromophore was extracted with *n*-butanol and absorbance was measured at 532 nm. The results were expressed as nmol MDA/g of *Artemia*.

### Culture of *A. franciscana* for Microinjection

Approximately, 1.5 g of brine shrimp cysts was hydrated in 89 ml of distilled water for 1 h. Sterile cysts and larvae were collected following decapsulation as described above. The cysts, suspended in 1-l glass bottles containing FASW, were incubated at 28°C for 28 h for hatching with a constant illumination of approximately 27 μE/m^2^ s. Hatched larvae at developmental stage instar II (stage at which mouth is open to ingest particles) were collected and transferred to 2-l glass bottles containing FASW. The larvae were fed daily with non-axenic green algae (*Tetraselmis suecica*) and grown to adults in a controlled temperature room (28°C) with a constant illumination of approximately 27 μE/m^2^ s (26).

### Preparation of dsRNA of *A. franciscana hsp70* and Green Fluorescence Protein (*gfp*)

The dsRNA specific to the *A. franciscana hsp70* (GenBank: AF427596.1) gene transcript was amplified (436 bp) using gene-specific primers (forward primer: 5′ GATGCAGGTGCCATTGC 3′ and reverse primer: 5′ AGCTCCTCAAAACGGGC 3′); each primer included the T7 promoter (5′ TAATACGACTCACTATAGGG 3′) at their 5′ ends. The PCR was performed at 94°C for 3 min, followed by 35 cycles of 94°C for 25 s, 53°C for 25 s, and 72°C for 2 min, and then the final extension at 72°C for 5 min. The purified PCR product was used as a template to generate dsRNA by using the MEGAscript RNAi kit (Ambion, USA), and the *in vitro* transcription reaction was performed according to the manufacturer’s instructions. Briefly, PCR-purified product was incubated overnight at 37°C with four ribonucleotides (ATP, CTP, GTP, and UTP), T7 enzyme mix, and reaction buffer. Then, the dsRNA was incubated at 37°C for 1 h with DNase I and RNAse for nuclease digestion to remove any template DNA and ssRNA that did not anneal. The *hsp70* dsRNA, quantified in Nanodrop spectrophotometer (ThermoFisher Scientific, Belgium), was subjected to 1.5% agarose gel electrophoresis in order to check the integrity and efficiency of duplex formation. In addition, a gfp fragment (455bp) was amplified to prepare dsRNA of *gfp*, which served as a negative control using primers (forward 5′ AGAGCGCTTCTCGTTGGGG 3′ and reverse 5′ AGACCTGAAGTTCATCTGC 3′); each primer included the T7 promoter (5′ TAATACGACTCACTATAGGG 3′) at their 5′ ends.

### Microinjection of *Artemia* Females With dsRNA

The dsRNA specific to *hsp70* and *gfp* was mixed separately in 1:10 ratio (v/v) with 0.5% phenol red in Dulbecco’s phosphate-buffered saline (Sigma-Aldrich). The diluted dsRNA either *hsp70* or *gfp* (250 nl of solution containing approximately 80 ng dsRNA) was injected with a FemtoJet^®^ microinjector (Eppendorf, USA) using Femtotips II microinjection capillary tips (Eppendorf, USA) to egg sacs of adult *Artemia* female while viewing under stereomicroscope (Figure S3 in Supplementary Material) (18). A total of thirty numbers of females were injected with dsRNA for each *hsp70* and *gfp*. Injected females were observed for 2 h, and the animals that retained dye, remained healthy, and could swim properly were employed for further experiments (Figure S4 in Supplementary Material). Mating pairs, i.e., injected adult female and male, were transferred to individual well in a 6-well plate containing 35 ppt artificial sea water. The *Artemia* pairs were fed daily with green algae, *T. suecica*, and maintained in a controlled temperature room (28°C) with a constant illumination of approximately 27 μE/m^2^ s. After 5 days, larvae were collected from each mating pairs (injected with dsRNA *hsp70* and *gfp*) for further analysis.

### Brine Shrimp Challenge Assay

The brine shrimp larvae obtained from a single female injected with dsRNA specific to either *hsp70* or *gfp* were collected, counted, and thereafter transferred to sterile 50-ml falcon tubes containing 30-ml FASW. The larvae were pretreated with an optimized dose of phloroglucinol for 2 h at 28°C. They were rinsed repeatedly with FASW to wash away the compound and then allowed to recover for 2 h at 28°C. Following recovery, the larvae were transferred to sterile 40-ml glass tubes containing 10 ml of FASW. Subsequently, the tubes were inoculated with *V. parahaemolyticus* MO904 strain at 10^7^ cells/ml. The survival of *Artemia* was scored 48 h after the addition of a pathogen. The brine shrimp larvae, obtained from either *hsp70* or *gfp* dsRNA-injected females, which did not receive phloroglucinol pretreatment, and either challenged with *V. parahaemolyticus* or not, served as negative and positive controls.

### Detection of *hsp70* mRNA and Hsp70 Protein in Brine Shrimp Larvae

The larvae released from *Artemia* females injected with either *hsp70* or *gfp* dsRNA were transferred to sterile 50-ml falcon tubes containing 30-ml FASW. The larvae were pretreated with an optimized dose of phloroglucinol for 2 h at 28°C. They were rinsed repeatedly with FASW to wash away the compound and then allowed to recover for 2 h at 28°C. Subsequently, the larvae were washed with sterile distilled water, counted (20 nos.), and collected in 1.5-ml sterile eppendorf tubes. The larvae were homogenized with a sterile microfuge pestle in 100 µl of TRIzol^®^ reagent (Invitrogen, USA) (15, 18). The homogenate was centrifuged at 12,000 × *g* for 10 min at 4°C, and the supernatant was transferred to new sterile eppendorf tubes prior to RNA isolation according to the manufacturer’s protocols. An equal amount of larvae RNA (determined by NanoDrop spectrophotometer, ThermoFisher Scientific, Belgium) was used for cDNA synthesis, using Superscript^®^ III First-Strand synthesis supermix for qRT-PCR kit (Invitrogen, USA). Subsequently, 5 µl of cDNA product was used for PCR amplification of *hsp70* using the forward and reverse primers (as described above). PCR products were resolved in 1.5% (w/v) agarose gel, stained with GelRed™ nucleic acid gel stain, and visualized by ChemiDoc MP imaging system (BioRad, Belgium).

For the detection of Hsp70 protein in larvae from females injected with dsRNA specific to either *hsp70* or *gfp*, the larvae were pretreated with an optimized dose of phloroglucinol as described above. Following pretreatment, 20 larvae (washed in sterile DW) were collected in 1.5-ml sterile eppendorf tubes. The larvae were homogenized in an equal volume of loading buffer (5 µl), vortexed, heated for 5 min at 95°C, and 15 µl of each sample was resolved in 10% SDS-PAGE gel (BioRad, Belgium) (15). Subsequently, Hsp70 protein is detected as described previously in Section “Protein Extraction and Detection of Hsp70 in Brine Shrimp Larvae.”

### Xenic Brine Shrimp Challenge Assay

Brine shrimp cysts were hatched in xenic (germ-associated) condition, following decapsulation and hatching procedures as described by Sorgeloos et al. (27) and Baruah et al. (21), and brine shrimp larvae were collected after 28 h, at developmental stage II. The dose–response relationship of phloroglucinol was performed as described previously by Ref. (22) with slight modification. Briefly, the hatched brine shrimp larvae were collected, counted volumetrically, and thereafter transferred to 50-ml falcon tubes containing 30-ml seawater. The larvae were pretreated with increasing doses of phloroglucinol (1, 2.5, 5, 10, 20, 30, 40, 50, and 100 µM) for 2 h at 28°C. The larvae were rinsed repeatedly with seawater to wash away the compound and then allowed to recover for 2 h. Following recovery period, a group of 20 larvae were transferred to 40-ml glass tubes containing 10 ml of seawater. Subsequently, the tubes were inoculated with *V. parahaemolyticus* MO904 strain at 10^7^ cells/ml, and the *Artemia* therein was fed with autoclaved LVS3 (10^7^ cells/ml). The survival of *Artemia* was scored 2 days after the addition of pathogen, and each treatment was done in quintuplicate.

### *Macrobrachium rosenbergii* Rearing System

The experiments were carried out at the Laboratory of Aquaculture & *Artemia* Reference Center, Ghent University, Belgium, in a controlled temperature room. Acclimatized adult freshwater shrimp (*M. rosenbergii*) obtained from laboratory and maintained in four separate freshwater recirculation units were used as brooders. For each experiment, larvae from single ovigerous female breeder were used. Matured female with fully ripe fertilized eggs (indicated by dark gray eggs) were transferred to the hatching tanks (30 l) containing brackish water (6 g/l salinity). Twenty-four hours after hatching, the larvae were collected and stocked in two separate brackish water (12 g/l salinity) recirculation units and fed with newly hatched axenic brine shrimp larvae. The broodstock management techniques were followed as previously described (28, 29).

### *Macrobrachium* Lethality Test and Challenge Assay

In total, four separate experiments were performed in a controlled temperature room. In the first experiment, it was determined whether *V. parahaemolyticus* MO904 strain could cause infection in *Macrobrachium* larvae, and to this end, a dose–response study was determined. A group of 10 *Macrobrachium* larvae (8 days old) were collected and transferred to 150-ml glass tubes containing 100-ml sterile brackish water (12 g/l salinity) (29). Subsequently, the larvae were exposed to four doses (10^4^, 10^5^, 10^6^, and 10^7^ cells/ml of total rearing water) of *V. parahaemolyticus* MO904 strain. *Macrobrachium* larvae that were not challenged with *V. parahaemolyticus* served as negative control. Survival was scored at 12, 24, 36, and 48 h after the addition of the pathogen. The *V. parahaemolyticus* dose-inducing mortality of prawn larvae near to 80% in 24 h was considered as an optimum infectious dose and selected for the experimental challenge.

In the second experiment, the cytotoxic effect of phloroglucinol was determined in *M. rosenbergii* larvae as previously described by Baruah et al. (13). The larvae were pretreated with an increasing concentration of phloroglucinol as described above in the *M. rosenbergii* dose–response study. Subsequently following recover period, toxicity assay was performed in sterile 150-ml glass tubes with 10 larvae in 100-ml sterile brackish water (12 g/l salinity) per tube. The toxicity of compound was determined after 12, 24, 36, and 48 h by scoring the number of survivors as previously described (13). The larvae that did not receive the phloroglucinol pretreatment served as negative control. Each treatment and control were performed in three replicates.

In third and fourth experiments, the protective effect of phloroglucinol was determined. In brief, *Macrobrachium* larvae (8 days old) were collected, counted, and transferred to 500-ml sterile glass bottle containing sterile brackish water (12 g/l salinity). The larvae were pretreated with increasing concentrations of phloroglucinol (1, 2.5, 5, 10, 20, and 30 µM) for 2 h at 28°C. They were rinsed repeatedly with brackish water to wash away the compound and then allowed to recover for 2 h at 28°C. Following the recovery period, a group of 10 larvae were transferred to sterile 150-ml glass tubes containing 100-ml sterile brackish water (12 g/l salinity). Subsequently, the tubes were inoculated with *V. parahaemolyticus* MO904 strain at 10^6^ cells/ml. The survival of larvae was scored 12, 24, 36, and 48 h after the addition of pathogen. *Macrobrachium* larvae that did not receive phloroglucinol pretreatment either challenged or not challenged with *V. parahaemolyticus* served as negative and positive controls.

### Detection of Hsp70 in *Macrobrachium* Larvae

Following pretreatment with phloroglucinol, live *Macrobrachium* larvae (0.1 g w/v) were rinsed in sterile distilled water, collected in 1.5-ml sterile eppendorf tubes, and immediately frozen in liquid nitrogen and stored in −80°C. The samples were homogenized and processed for protein extraction as described above. The protein concentration was determined by the Bradford method (24) using bovine serum albumin as standard. The supernatant samples of *Macrobrachium* larvae were then electrophoresed in 10% SDS-PAGE gel (BioRad, Belgium) and transferred to polyvinylidene fluoride membrane (BioRad Immuno-Blot™ PVDF) for antibody probing to detect Hsp70 protein as described previously in the above section.

### Statistical Analysis

Survival data were arcsin transformed to satisfy normality and homoscedasticity requirements as necessary. The data were then subjected to one-way analysis of variances (ANOVA) followed by Duncan’s multiple range test using statistical software statistical package for the social sciences version 24.0. The effect of RNAi on survival of brine shrimp larvae was analyzed by two-way ANOVA. *P*-values of ≤0.001 were considered significant. Survival data of *M. rosenbergii* were subjected to logistic regression analysis using GenStat 16 (VSN International, Hemel Hempstead, UK) to determine significant differences between the control and the treatment.

## Results

### Phloroglucinol Pretreatment of Axenic Brine Shrimp Larvae Protects Them Against Subsequent *V. parahaemolyticus* Challenge and Is Associated With Hsp70 Induction

**Figure 1:**
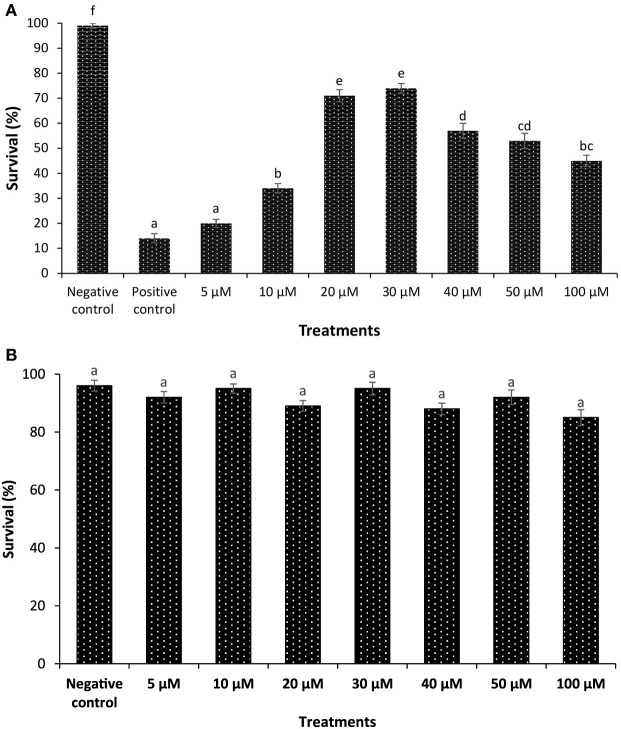

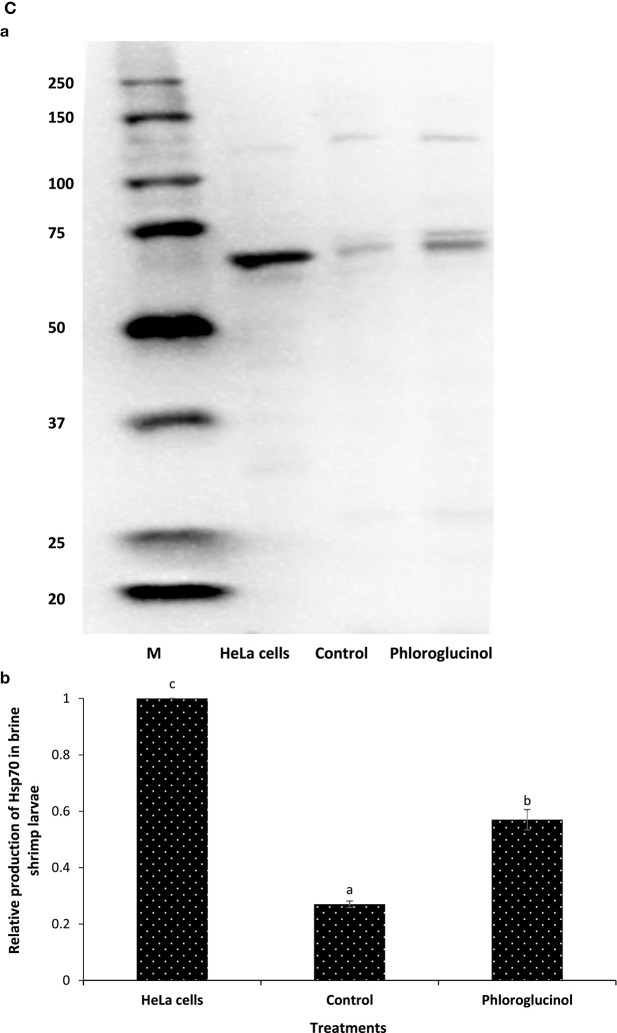
Phloroglucinol pretreatment of axenic brine shrimp larvae protects them against subsequent *Vibrio parahaemolyticus* challenge and is associated with heat shock protein (Hsp70) induction. **(A)** Survival (%) of phloroglucinol-pretreated brine shrimp larvae after 48 h of challenge with *V. parahaemolyticus* MO904. The larvae were pretreated with phloroglucinol at the indicated doses for 2 h, rinsed to wash away the compound, and then allowed to recover for 2 h. The larvae were subsequently challenged with *V. parahaemolyticus* at 10^7^ cells/ml of rearing water. Non-pretreated larvae that were either challenged with *V. parahaemolyticus* (positive control) or unchallenged (negative control) served as controls. Error bars represent the standard error of five replicates; different letters indicate significant differences (*P* < 0.001). **(B)** Toxicity of phloroglucinol to gnotobiotic brine shrimp larvae. The larvae were pretreated with phloroglucinol at the indicated doses for 2 h, rinsed to wash away the compound, and then allowed to recover for 2 h. Non-pretreated larvae served as negative controls. Survival was recorded 48 h after the recovery period. Error bars represent the standard error of five replicates; different letters indicate significant differences (*P* < 0.001). **(C)** Induction of Hsp70 protein in brine shrimp larvae pretreated with phloroglucinol (30 µM). Non-pretreated larvae were maintained as control. (a) Protein extracted from control and treatment groups was resolved on an SDS-PAGE gel and then transferred to a polyvinylidene fluoride membrane and probed with an antibody to brine shrimp Hsp70. Molecular mass standards (M) in kilodaltons (protein ladder) are shown on the left. The induction of Hsp70 in HeLa cells was regarded as 1. (b) Quantitative analysis of Hsp70 levels in brine shrimp larvae is presented relative to Hsp70 production in HeLa cells. Error bars represent the standard error of three replicates; different letters indicate significant differences (*P* < 0.001).

In the first experiment, we investigated whether phloroglucinol could confer protection to the host *Artemia* against *V. parahaemolyticus* MO904 strain, which can cause AHPND in shrimp (Figure S5 in Supplementary Material). We found that brine shrimp larvae that received phloroglucinol pretreatment in the range of 5–100 µM exhibited a significant increase in the survival compared to positive control. However, the maximum survival (11-folds or more) was observed at a concentration of 30 µM (Figure 1A). By contrast, the survival of brine shrimp larvae pretreated with phloroglucinol higher than 30 µM appeared to decrease. Therefore, we next investigated whether it was due to a toxic effect of phloroglucinol. Brine shrimp larvae exposed with phloroglucinol in the range of 5–100 µM did not exhibit any significant difference in survival when compared with unexposed, control larvae (Figure 1B). This result indicates that phloroglucinol appeared to be nontoxic to the experimental animal under the present experimental conditions. Next, to determine whether the induction of Hsp70 may be a mechanism supporting the resistance of phloroglucinol-treated brine shrimp larvae against *V. parahaemolyticus* challenge, we analyzed the temporal induction profile of Hsp70 *in vivo*, since the role of inducible Hsp70 in eliciting protective immunity against bacterial infection has been previously described (12, 13, 17, 20, 30, 31). Interestingly, we found that phloroglucinol pretreatment significantly increased Hsp70 production (twofolds or more) in brine shrimp larvae as compared with the control (Figure 1C). This result suggests that phloroglucinol is an effective inducer of Hsp70 and the protective effect of phloroglucinol is paralleled with the Hsp70 induction.

### Phloroglucinol-Generated Prooxidant Activity Mediates the Protective Response in Brine Shrimp Larvae

**Figure 2:**
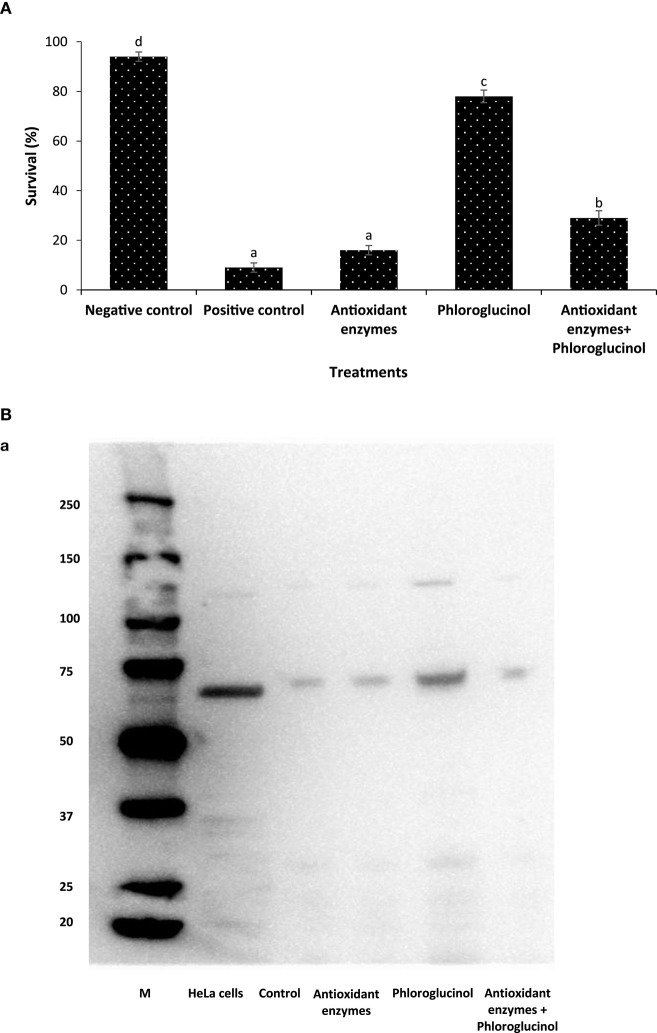

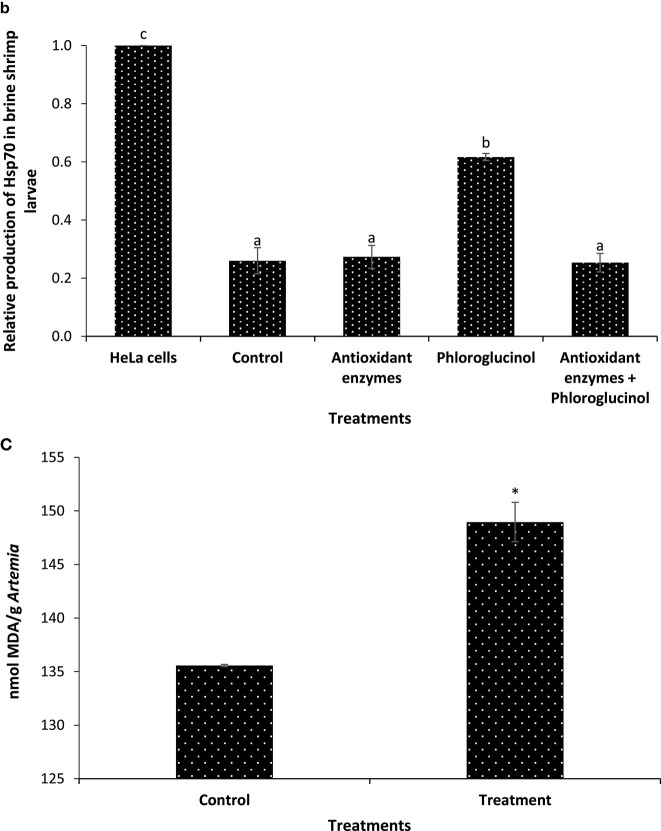
Phloroglucinol-generated prooxidant activity mediates the protective response in brine shrimp larvae. **(A)** Survival (%) of brine shrimp larvae pretreated with antioxidant enzymes, phloroglucinol, or in combination with both (antioxidant enzymes and phloroglucinol) after 48 h of challenge with *Vibrio parahaemolyticus* MO904. The larvae were pretreated with antioxidant enzymes (catalase, 10 mg/l and superoxide dismutase (SOD), 75 units), phloroglucinol (30 µM), and mixture of antioxidant enzymes and phloroglucinol for 2 h, rinsed to wash away the compound, and then allowed to recover for 2 h. The larvae were subsequently challenged with *V. parahaemolyticus* at 10^7^ cells/ml of rearing water. Non-pretreated larvae that were either challenged with *V. parahaemolyticus* (positive control) and those not challenged with *V. parahaemolyticus* (negative control) served as controls. Error bars represent the standard error of five replicates; different letters indicate significant differences (*P* < 0.001). **(B)** Induction of heat shock protein (Hsp70) protein in brine shrimp larvae. The brine shrimp larvae were pretreated with antioxidant enzymes, phloroglucinol, or in combination with both (antioxidant enzymes and phloroglucinol) as described in Figure 2A. Non-pretreated larvae were maintained as control. (a) Protein extracted from different treatment groups was resolved on an SDS-PAGE gel and then transferred to a polyvinylidene fluoride membrane and probed with an antibody to brine shrimp Hsp70. Molecular mass standards (M) in kilodaltons are shown on the left. The induction of Hsp70 in HeLa cells was regarded as 1. (b) Quantitative analysis of Hsp70 levels in the brine shrimp larvae is presented relative to Hsp70 production in HeLa cells. Error bars represent the standard error of three replicates; different letters indicate significant differences (*P* < 0.001). **(C)** Effect of phloroglucinol on lipid peroxidation in brine shrimp larvae. Lipid peroxidation is expressed as malondialdehyde (MDA) content in each sample. The larvae were pretreated with phloroglucinol (30 µM) for 2 h, rinsed to wash away the compound, and then allowed to recover for 2 h. The larvae were subsequently collected and analyzed for MDA/g of *Artemia*. Non-pretreated larvae were maintained as control. Error bars represent the standard error of two independent samples; asterisks represent significant difference between the control and the treatment, *P < 0.001.

We next sought to investigate the mechanism of action of phloroglucinol in inducing Hsp70 within *Artemia*. Since reactive oxygen species (e.g., hydrogen peroxide, H_2_O_2_) generated from the polyphenolic compounds has been linked with Hsp70 induction (17, 32), we hypothesized that phloroglucinol induced Hsp70 by the mechanism of generating ROS and that eliminating these molecules by using ROS-scavenging enzymes, the phloroglucinol-mediated Hsp70 induction can be neutralized. We verified these findings by two alternative approaches. We found that brine shrimp larvae pretreated with phloroglucinol showed a significant increase in the survival and Hsp70 production (Figures 2A,B). However, co-pretreatment of larvae with phloroglucinol and antioxidant enzyme mixture resulted in a significant decrease in the survival and Hsp70 production as compared to the larvae pretreated with phloroglucinol (Figures 2A,B). We also further examined the prooxidant characteristics (i.e., generation of ROS: hydrogen peroxide, H_2_O_2_) of the phloroglucinol through lipid peroxidation by analyzing MDA content in the phloroglucinol-treated brine shrimp larvae. We found that pretreated larvae have significantly higher MDA content/g *Artemia* when compared with the negative control larvae (Figure 2C). These results indicate that prooxidant activity generated by phloroglucinol in the *Artemia* appeared to be, at least in part, involved in the induction of Hsp70 within brine shrimp larvae.

### Knockdown of Hsp70 by RNAi Reduces the Tolerance of Brine Shrimp Larvae to *V. parahaemolyticus* Infection

**Figure 3:**
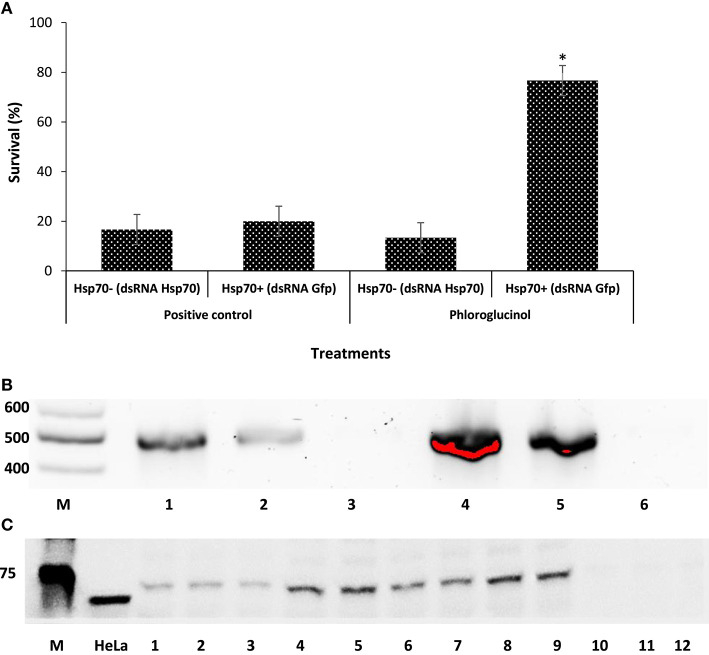
Knockdown of heat shock protein 70 (Hsp70) by RNA interference (RNAi) reduces the tolerance of brine shrimp larvae to *Vibrio parahaemolyticus* infection. **(A)** Hsp70 protects brine shrimp larvae against *V. parahaemolyticus* MO904. Brine shrimp larvae obtained from females injected with either *gfp* (+Hsp70) or *hsp70* dsRNA (−Hsp70) were pretreated with phloroglucinol (30 µM) for 2 h, rinsed to wash away the compound, and then allowed to recover for 2 h. The larvae were subsequently challenged with *V. parahaemolyticus* at 10^7^ cells/ml. Phloroglucinol non-pretreated larvae with and without *hsp70* that were challenged with *V. parahaemolyticus* served as positive control. Error bars represent the standard error of six replicates; asterisks represent significant difference between the control and the treatment, *P < 0.001. **(B)** Knockdown of *hsp70* mRNA in brine shrimp larvae. Equal amounts of RNA from brine shrimp larvae were amplified by RT-PCR and the products were resolved by electrophoresis in agarose gels. M—100 bp DNA ladder, Lane 1—wild-type brine shrimp larvae, Lane 2—brine shrimp larvae injected with dsRNA *gfp*, Lane 3—brine shrimp larvae injected with dsRNA *hsp70*, Lane 4—wild-type brine shrimp larvae pretreated with phloroglucinol, Lane 5—brine shrimp larvae injected with dsRNA *gfp* and pretreated with phloroglucinol, Lane 6—brine shrimp larvae injected with dsRNA *hsp70* and pretreated with phloroglucinol. **(C)** Knockdown of Hsp70 protein in brine shrimp larvae. The brine shrimp larvae from females injected with dsRNA for either *gfp* or *hsp70* were resolved by electrophoresis in SDS polyacrylamide gels, blotted to nitrocellulose, and probed with Hsp70 antibody (3A3). The secondary antibody was HRP-conjugated donkey anti-mouse IgG. M—Protein ladder in kilodaltons, Lanes 1–3—wild-type brine shrimp larvae, Lanes 4–6—wild-type brine shrimp larvae pretreated with phloroglucinol, Lanes 7–9—brine shrimp larvae injected with dsRNA *gfp* and pretreated with phloroglucinol, Lanes 10–12—brine shrimp larvae injected with dsRNA *hsp70* and pretreated with phloroglucinol.

There exists a correlation between the amount of induced Hsp70 and the degree of improved protective response against *V. parahaemolyticus*. To gain insight into the molecular bases for the observed effects, we used RNAi to assess the role of Hsp70 in protecting brine shrimp larvae against *V. parahaemolyticus* MO904 strain. As RNAi-mediated gene-silencing technique has been very frequently used to investigate the function of critical genes involved in the immune response (18, 33–36), we have prepared dsRNA specific to Hsp70 and injected *A. franciscana* females in order to eliminate Hsp70 mRNA and protein from larvae released from these females. At first, we have examined the effect of Hsp70 knockdown on the survival of larvae released from females injected with either *hsp70* dsRNA or *gfp*. We found that larvae lacking or containing Hsp70 survived equally after being released from injected females and Hsp70 knockdown did not affect the viability of brine shrimp larvae. Furthermore, our analysis revealed that phloroglucinol pretreatment to larvae collected from females injected with either *hsp70* dsRNA (−Hsp70) or *gfp* (+Hsp70) showed a significant difference in the survival against *V. parahaemolyticus* (Figure 3A). The survival was significantly increased (~6-folds) in the pretreated larvae containing Hsp70 when compared with larvae lacking Hsp70 (Table S1). We therefore reasoned that the difference in the survival of larvae might be due to the difference in the expression of Hsp70. To test the hypothesis that dsRNA may have knockdown *hsp70* mRNA and protein, we have analyzed *hsp70* mRNA and Hsp70 protein in *A. franciscana* larvae collected from females injected with dsRNA. We found that females injected with *hsp70* dsRNA knockdown the *hsp*70 mRNA in larvae (Figure 3B). Furthermore, the Western blot analysis of Hsp70 protein showed that Hsp70 was absent in the larvae released by females injected with dsRNA *hsp70*, but not in the larvae from females injected with *gfp* dsRNA (Figure 3C). These results therefore confirm that phloroglucinol-mediated Hsp70 production plays an essential role in providing protection against *V. parahaemolyticus* MO904 strain.

### Pretreatment of Xenic Brine Shrimp Larvae and *Macrobrachium* Larvae With Phloroglucinol Protects Them Against Subsequent *V. parahaemolyticus* Challenge

**Figure 4:**
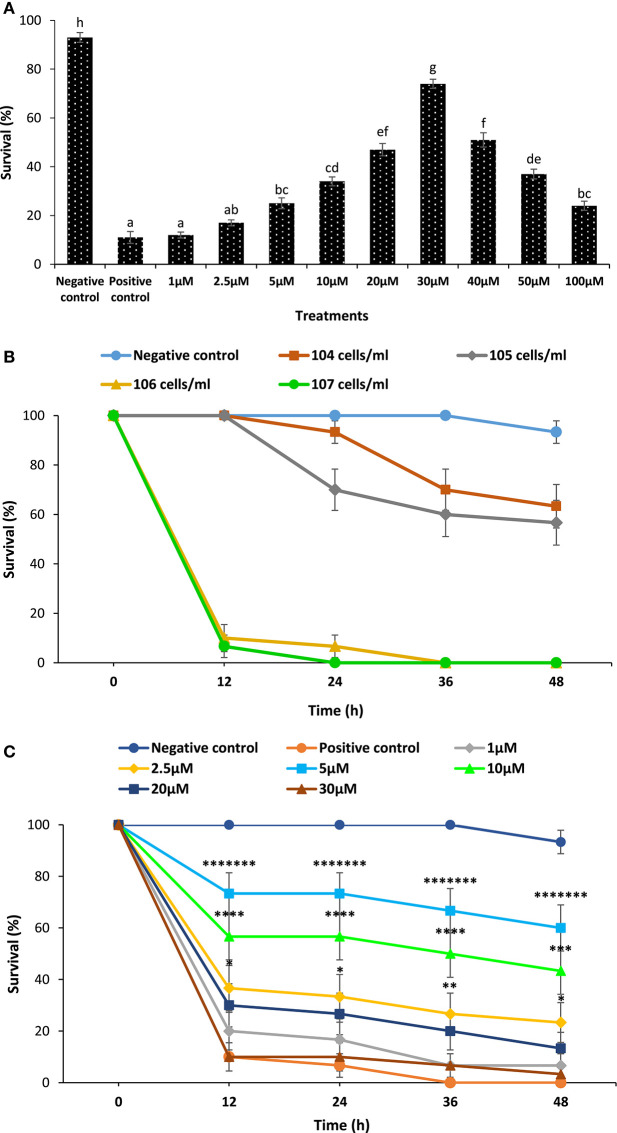

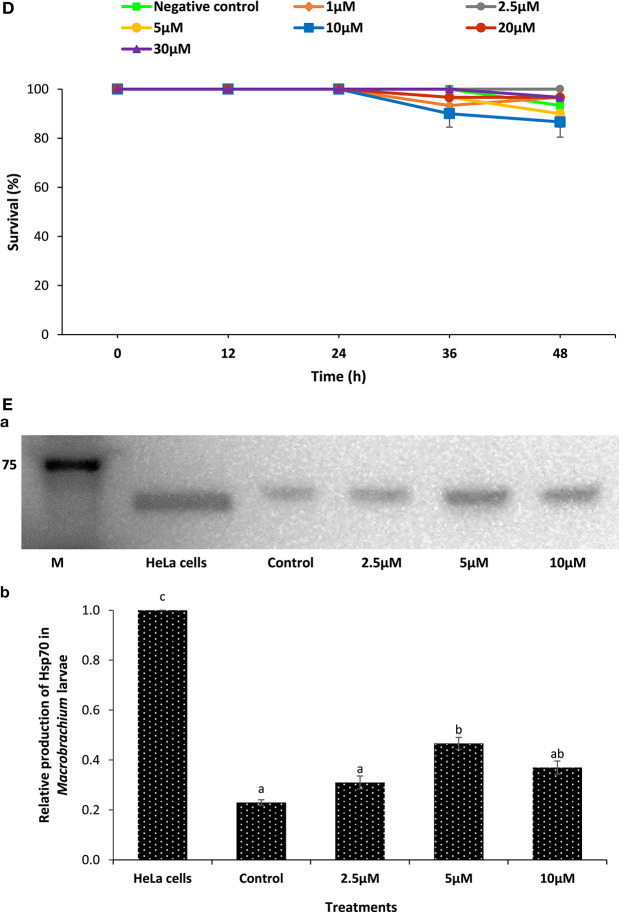
Pretreatment of xenic brine shrimp larvae and *Macrobrachium* larvae with phloroglucinol protects them against subsequent *Vibrio parahaemolyticus* challenge. **(A)** Survival (%) of phloroglucinol-pretreated xenic brine shrimp larvae after 48 h of challenge with *V. parahaemolyticus* MO904. The larvae were pretreated with phloroglucinol at the indicated doses for 2 h, rinsed to wash away the compound, and then allowed to recover for 2 h. The larvae were subsequently challenged with *V. parahaemolyticus* at 10^7^ cells/ml of rearing water. Non-pretreated larvae that were either challenged with *V. parahaemolyticus* (positive control) or unchallenged (negative control) served as controls. Error bars represent the standard error of five replicates; different letters indicate significant differences (*P* < 0.001). **(B)** Proportion of survived of *Macrobrachium* larvae (8 days old) after 12, 24, 36, and 48 h post challenge with *V. parahaemolyticus* MO904 strain. Four infectious doses (10^4^, 10^5^, 10^6^, and 10^7^ cells/ml) were tested in the *Macrobrachium* larvae. The *V. parahaemolyticus* dose provoking significant mortality near to 80% at 24 h in *Macrobrachium* larvae was considered an optimum dose and selected for the experimental challenge. The unchallenged was served as negative controls. Values are presented as mean ± SE (*n* = 3). **(C)** The effect of phloroglucinol pretreatment on the survival of *Macrobrachium* larvae (8 days old) after 12, 24, 36, and 48 h post challenge with *V. parahaemolyticus*. The larvae were pretreated with phloroglucinol at the indicated doses for 2 h, rinsed to wash away the compound, and then allowed to recover for 2 h. The *Macrobrachium* larvae were subsequently challenged with *V. parahaemolyticus* at 10^6^ cells/ml of rearing water. Non-pretreated larvae that were either challenged with *V. parahaemolyticus* (positive control) and those not challenged with *V. parahaemolyticus* (negative control) served as controls. Values are presented as mean ± SE (*n* = 3). Asterisks represent significant difference between the control and the treatment. *P < 0.05, **P < 0.01, ***P < 0.001, ****P < 0.0001, *****P < 0.00001, ******P < 0.000001, *******P < 0.0000001. **(D)** Toxicity of phloroglucinol to *Macrobrachium* larvae (8 days old). The larvae were pretreated with phloroglucinol at the indicated doses for 2 h, rinsed to wash away the compound, and then allowed to recover for 2 h. Non-pretreated larvae served as controls. Survival was recorded 48 h after the recovery period. Values are presented as mean ± SE (*n* = 3). **(E)** Induction of Hsp70 protein in *Macrobrachium* larvae (8 days old) pretreated with phloroglucinol at indicated doses. Non-pretreated larvae were maintained as control. (a) Protein extracted from different treatment groups was resolved on an SDS-PAGE gel and then transferred to a polyvinylidene fluoride membrane and probed with an antibody to brine shrimp Hsp70. Molecular mass standards (M) in kilodaltons are shown on the left. The induction of Hsp70 in HeLa cells was regarded as 1. (b) Quantitative analysis of Hsp70 levels in the brine shrimp larvae is presented relative to Hsp70 production in HeLa cells. Error bars represent the standard error of three replicates; different letters indicate significant differences (*P* < 0.001).

To further confirm the contribution of phloroglucinol on the survival of xenic brine shrimp and *Macrobrachium* larvae against *V. parahaemolyticus*, we conducted additional *in vivo* survival assay as described in Figure 4. We assumed that phloroglucinol imparts protection to xenic brine shrimp larvae against *V. parahaemolyticus* infection and does not have any significant difference when compared with gnotobiotic brine shrimp larvae. Second, we have used *M. rosenbergii* system to further verify that phloroglucinol mediates the production of Hsp70 and induces resistance in *Macrobrachium* larvae against pathogenic *V. parahaemolyticus* MO904 strain.

### *In Vivo* Confirmation in Xenic Brine Shrimp Larvae

We investigated whether phloroglucinol contributes to the induction of protective response in xenic brine shrimp larvae against *V. parahaemolyticus* MO904 strain. In agreement with the gnotobiotic brine shrimp larvae survival results, we found that xenic brine shrimp larvae pretreated with varying concentrations of phloroglucinol protect larvae when subsequently challenged with pathogenic *V. parahaemolyticus* (Figure 4A). Moreover, the survival was recorded maximum (six times or more) at a concentration of 30 µM when compared with the control larvae. The phloroglucinol pretreatment at a concentration higher than 30 µM did not further improve the larvae survival. By contrast, survival tended to decrease with further increase in the concentration.

### Further Validation in *M. rosenbergii*

In the validation experiment, the efficiency of phloroglucinol to protect *Macrobrachium* larvae from *V. parahaemolyticus* infection was investigated. At first, we have examined the optimum dose of *V. parahaemolyticus* MO904 strain required for the dose–response study. We found that *M. rosenbergii* post larvae (8 days old) when exposed to four infectious doses of *V. parahaemolyticus* (10^4^, 10^5^, 10^6^, and 10^7^ cells/ml) showed a significant difference in the survival of larvae (Figure 4B). The *V. parahaemolyticus* infectious dose, 10^6^ cells/ml, provoking a significant mortality near to 80% at 24 h, was considered an optimum dose and selected for the experimental challenge.

Earlier in the study, it is shown that phloroglucinol induces the Hsp70 production and protects brine shrimp larvae from *V. parahaemolyticus* infection. This has led to the suggestion that phloroglucinol might have a protective response in other shrimp species, e.g., freshwater shrimp, *M. rosenbergii*. To test the hypothesis, we first investigated whether phloroglucinol pretreatment has a significant role in the survival of *Macrobrachium* larvae, and we found that larvae pretreated with phloroglucinol showed a significant increase (sevenfolds or more) in the survival as compared to the control (positive control) (Figure 4C). We have also investigated whether the phloroglucinol has some toxic effect on the larvae of *Macrobrachium*, since the survival of larvae pretreated above 5 µM phloroglucinol concentration appeared to decrease. We found that phloroglucinol pretreatment does not have any toxic effect on the experimental animals (Figure 4D). In the next experiment, we examined whether an observed increase in the survival of larvae is related to the increase in the Hsp70 production. We found that Hsp70 production increased significantly (twofolds or more) in the *Macrobrachium* larvae pretreated with phloroglucinol as compared to the control (Figure 4E). These results indicate that phloroglucinol stimulates the Hsp70 production in larvae, which may be involved in eliciting immune response and protects *Macrobrachium* larvae from pathogenic *V. parahaemolyticus* infection.

## Discussion

The bacterial species, *V. parahaemolyticus* and especially AHPND-causing strains, such as MO904 are a major cause of infectious disease and mortality in wild and cultured shrimp species (1, 3). The AHPND strains of *V. parahaemolyticus* after acquiring a plasmid (pVA1) (63–70 kb) express deadly toxin PirA^VP^ and PirB^VP^ that damages the shrimp tissue, primarily the hepatopancrease (Hp) R (resorptive) cells, B (blister) cells, F (fibrillar) cells, and E (embryonic cells), resulting in the shrinkage of Hp tubular epithelial cells and sloughing of Hp tubular lumen (36). The larval stages of shrimp species, in general, are more susceptible to the pathogenic *V. parahaemolyticus*, and the infection develops quickly, starting approximately after 8 days of post-larvae stocking, and severe mortalities occur during the first 20–30 days of culture (37). This motivated us to develop an infection model for AHPND using gnotobiotic *Artemia*, and we found that *V. parahaemolyticus* MO904 strain indeed could cause mortality in axenic *Artemia*. Subsequent studies were carried out to validate these findings under xenic condition, and results showed that MO904 strain was virulent to both xenic *Artemia* and *Macrobrachium* larvae. However, it remains to be established whether the mortality is PirA^VP^ and PirB^VP^ mediated. It would especially be of interest to establish which cells would be affected by PirA^VP^ and PirB^VP^.

The ability to stimulate defense system of invertebrate is considered to be central to control the pathogenesis of many microbial pathogens (17). In case of brine shrimp that lacks acquired immune system and depends on innate immune factors to build up resistance against microbial pathogens (like other invertebrate species), eliciting innate immune response might be a potential preventive modality for the control of biotic and abiotic stressors in aquaculture production system (33, 34, 38–41). Previous works have shown that the ability of natural/plant-based compounds to increase the resistance of host against bacterial infection is probably facilitating enhanced immune response (12, 20, 27, 38). Although the functional significance of natural or plant-based compounds in providing protection to *Artemia* against bacterial infection by a mechanism of inducing Hsp70 is reported in these studies, the role of natural/plant-based compounds against *V. parahaemolyticus* and the molecular mechanism behind the protective effect in host remain to be established. In this study, using gnotobiotic *Artemia* model system, it was shown that phloroglucinol, a polyphenol plant-based compound, isolated from marine brown algae belonging to the family Laminariaceae, containing 1,3,5-trihydroxy benzene as the basic moiety (42), provides protection to brine shrimp larvae against *V. parahaemolyticus* MO904 strain.

The ability of polyphenol plant-based compounds to induce resistance in host and prevent the microbial infection is functionally dependent on antioxidant property, prooxidant activity, and antimicrobial effects (12, 43, 44). Our results indicate that pretreatment of phloroglucinol exerted a protective effect in brine shrimp larvae against *V. parahaemolyticus* by mechanism of its prooxidant activity (e.g., generation of hydrogen peroxide, H_2_O_2_) (Figures 2A–C). In fact, such protective effect of phloroglucinol was not observed if the generation of reactive oxygen species was counteracted by the addition of antioxidant enzyme mixture (SOD and catalase) (Figure 2A) (17). In addition, we have also provided evidence for a link between the phloroglucinol prooxidant action and an increase in the Hsp70 protein levels, which might stimulate the immune response instigating resistance against *V. parahaemolyticus* in the brine shrimp larvae. This assumption is supported by the observation that the protective phenomenon was not observed when the induction of Hsp70 was inhibited by the ROS-scavenging enzymes mixture (Figures 2A,B) and consistent with the known beneficial roles that Hsp70 plays in defining the tolerance of organism to bacterial infection by eliciting the protective immunity (33, 45, 46).

However, phloroglucinol-mediated Hsp70 induction may represent only one of the possible underlying mechanisms. Hence, we used RNAi-mediated gene-silencing technique, to unravel the molecular mechanism behind the observed protective effect of the compound in protecting the brine shrimp larvae against *V. parahaemolyticus* (16, 18, 36). Our results indicate that increased survival in brine shrimp larvae pretreated with phloroglucinol is most likely due to a significant increase in Hsp70 production (Figures 3A–C). This is due to the fact that such protective effect of phloroglucinol was not observed if the adult *Artemia* females were injected with *hsp70* dsRNA. Therefore, our result provides here new insight on the mode of Hsp70-inducing action of phloroglucinol, in which the initial generation of prooxidant activity by the compound plays a key role in Hsp70 induction and protection of brine shrimp larvae against *V. parahaemolyticus* infection.

As for the gnotobiotic *A. franciscana*, we found that phloroglucinol has a protective response against *V. parahaemolyticus*. This led to postulate us that phloroglucinol might be contributing in the protection of xenic brine shrimp larvae and other shrimp species (e.g., *M. rosenbergii*) against *V. parahaemolyticus*. However, it is hard to generalize from the results of gnotobiotic *A. franciscana* alone because, first under germ-associated condition, the physiological response (increased prooxidant activity and Hsp70 production) in the host due to exposure of tested compound could simply reflect the influence of various microbial communities (LPS or other cellular component) rather than a genuine biological effect of the compound (46, 47). Second, it may be possible that the protective effect of phloroglucinol might be different in another host–pathogen model (i.e., *M. rosenbergii*–*V. parahaemolyticus*). Therefore, to address this question, we have analyzed xenic brine shrimp larvae and *Macrobrachium* larvae response against phloroglucinol and *V. parahaemolyticus*. We found that xenic brine shrimp larvae pretreated with phloroglucinol displayed a significantly increased survival of larvae against pathogenic *V. parahaemolyticus* infection (Figure 4A), and in agreement with gnotobiotic *A. franciscana* results, we therefore suggest that phloroglucinol induces resistance in brine shrimp larvae in both gnotobiotic and germ-associated conditions against *V. parahaemolyticus*. Furthermore, in case of *M. rosenbergii*, we provided evidence that phloroglucinol exerted a protective effect against *V. parahaemolyticus* by the induction of Hsp70, which might be involved in eliciting immune response in the host (Figures 4C,E) (32, 48, 49).

In conclusion, the results presented here provide new insight on the mode of action of phloroglucinol, as a novel inducer of prooxidant activity and Hsp70. Our finding also provides strong evidences that phloroglucinol-mediated Hsp70 production seems to be responsible for generating resistance against a pathogenic *V. parahaemolyticus* MO904 strain that can cause AHPND in shrimps. The ability of phloroglucinol to boost immunity and induce resistance in *A. franciscana* and *M. rosenbergii* makes phloroglucinol a potent biocontrol agent that may be valuable to avert *V. parahaemolyticus* infection in other shrimp species. The results obtained add new information about the mode of action of phloroglucinol and advance our knowledge of this compound as a potential disease-mitigating agent.

## Author Contributions

VK, KB, and PB conceived and designed the experiment. VK performed the experiments and drafted the figures and manuscript. VK, DN, EV, and GS made the laboratory analysis, statistics, and interpreted data. The manuscript is reviewed and edited by VK, KB, and PB. All the authors approved the final manuscript.

## Conflict of Interest Statement

The authors declare that the research was conducted in the absence of any commercial or financial relationships that could be construed as a potential conflict of interest.
