# Corrigendum: Phloroglucinol-Mediated Hsp70 Production in Crustaceans: Protection against *Vibrio parahaemolyticus* in *Artemia franciscana* and *Macrobrachium rosenbergii*

**DOI:** 10.3389/fimmu.2020.01860

**Published:** 2020-08-18

**Authors:** Vikash Kumar, Kartik Baruah, Dung Viet Nguyen, Guy Smagghe, Els Vossen, Peter Bossier

**Affiliations:** ^1^Laboratory of Aquaculture & Artemia Reference Center, Department of Animal Sciences and Aquatic Ecology, Faculty of Bioscience Engineering, Ghent University, Ghent, Belgium; ^2^ICAR-Central Inland Fisheries Research Institute (CIFRI), Barrackpore, India; ^3^Department of Crop Protection, Faculty of Bioscience Engineering, Ghent University, Ghent, Belgium; ^4^Laboratory of Animal Nutrition and Animal Product Quality, Department of Animal Sciences and Aquatic Ecology, Faculty of Bioscience Engineering, Ghent University, Ghent, Belgium

**Keywords:** gnotobiotic *Artemia*, *Vibrio parahaemolyticus*, phloroglucinol, heat shock protein 70, *Macrobrachium rosenbergii*

In the original article, there was a mistake in Figure 1A as published. We have recently discovered that incorrect brine shrimp survival data at different phloroglucinol doses were presented in the published paper. The corrected Figure 1A appears below.

**Figure 1:**
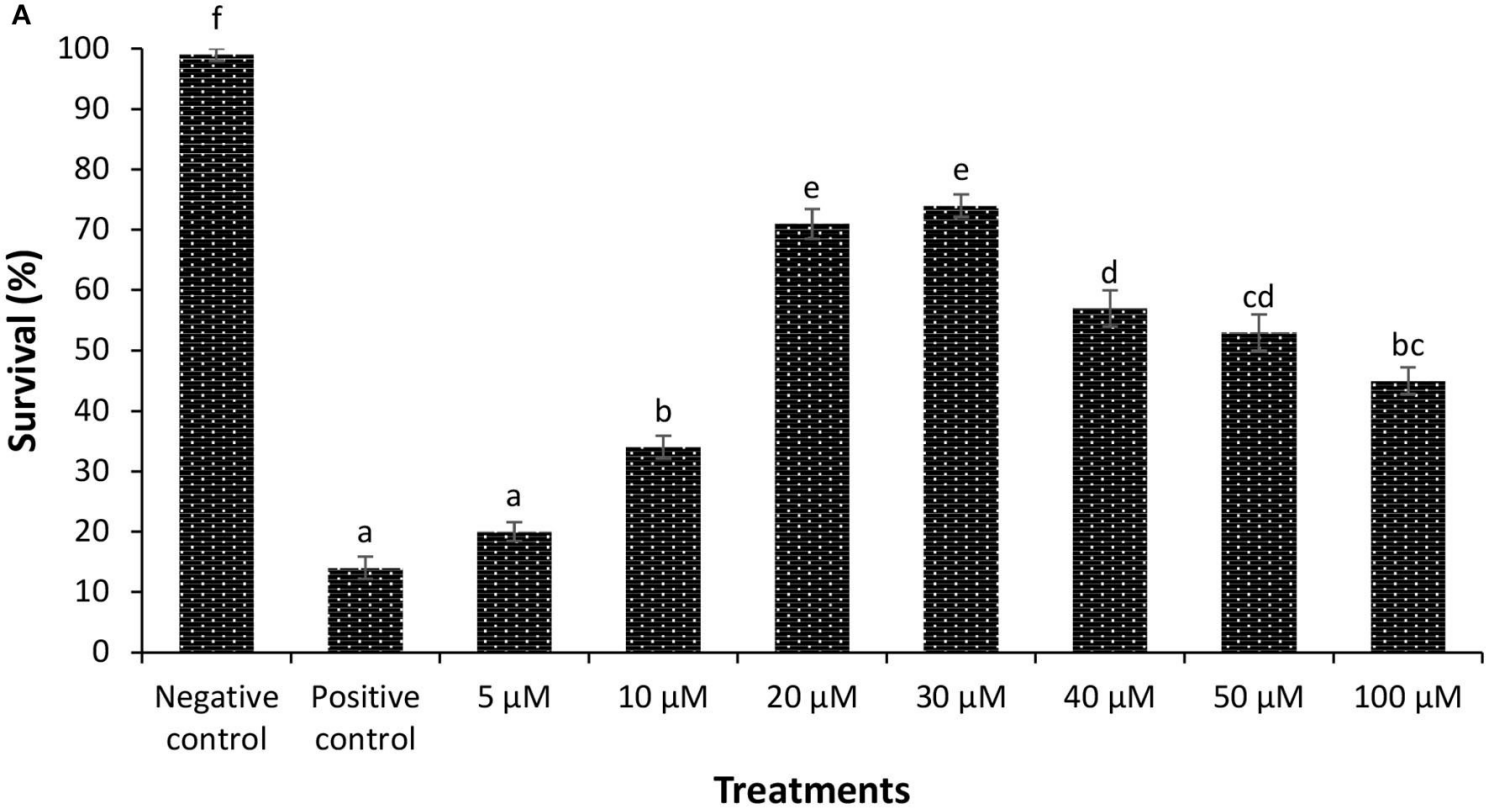
**(A)** Survival (%) of phloroglucinol-pretreated brine shrimp larvae after 48 h of challenge with *V. parahaemolyticus* MO904. The larvae were pretreated with phloroglucinol at the indicated doses for 2 h, rinsed to wash away the compound, and then allowed to recover for 2 h. The larvae were subsequently challenged with *V. parahaemolyticus* at 10^7^ cells/ml of rearing water. Non-pretreated larvae that were either challenged with *V. parahaemolyticus* (positive control) or unchallenged (negative control) served as controls. Error bars represent the standard error of five replicates; different letters indicate significant differences (*P* < 0.001).

The authors apologize for this error and state that this does not change the scientific conclusions of the article in any way. The original article has been updated.

