# Supplementary material for: Phloroglucinol-Mediated Hsp70 Production in Crustaceans: Protection against Vibrio parahaemolyticus in Artemia franciscana and Macrobrachium rosenbergii
Source: Front Immunol. 2018 May 22;9:1091. doi: 10.3389/fimmu.2018.01091 (PMC5972194; doi:10.3389/fimmu.2018.01091)
Supplement: Supplementary file 1 [file Data_Sheet_1.docx]

**SUPPORTING INFORMATIONS**


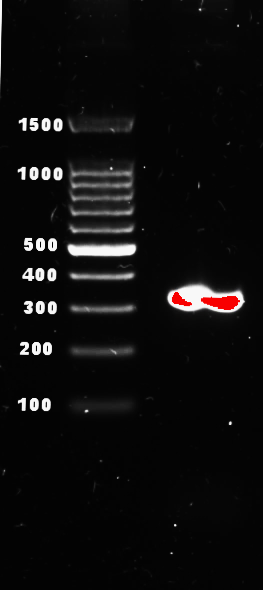


**1500**

**1000**

**500**

**400**

**300**

**200**

**100**

**~336 bp**

**M 1**

Figure S1 **Agarose gel of PCR amplicon from *Vibrio parahaemolyticus* MO904 strain using AP3 method**. M - 100 bp DNA ladder, Lane 1 – *V. parahaemolyticus* MO904 strain. Positive amplicon (~336 bp) for VP_AHPND_ bacteria from *V. parahaemolyticus* MO904 strain template DNA.

Figure S2 **Survival of brine shrimp larvae after 48 h of challenge with V. parahaemolyticus KM, MO605, RY, MO904, MO903 and CAMI170 strains***.* The *V. parahaemolyticus* MO904 strain induce significant mortality near to 80 % after 48 h in brine shrimp larvae was selected for the experimental challenge. The unchallenged was served as negative controls. Error bars represent the standard error of five replicates; different letters indicate significant differences (P < 0.001).


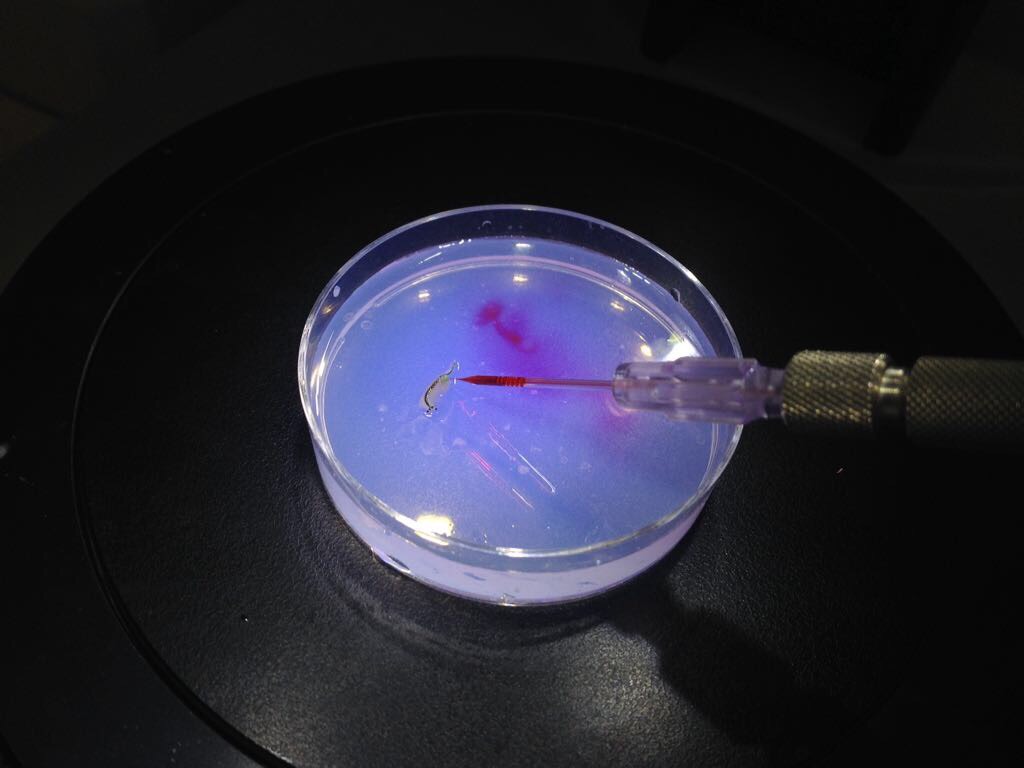


Figure S3 **Micro-injection of adult *Artemia* females with dsRNA.** The dsRNA either *hsp70* or *gfp* (250 nl of solution containing approximately 80 ng dsRNA) was injected with a FemtoJet^®^ microinjector using Femtotips II microinjection capillary tips to egg sacs of adult *Artemia* female while viewing under stereomicroscope.


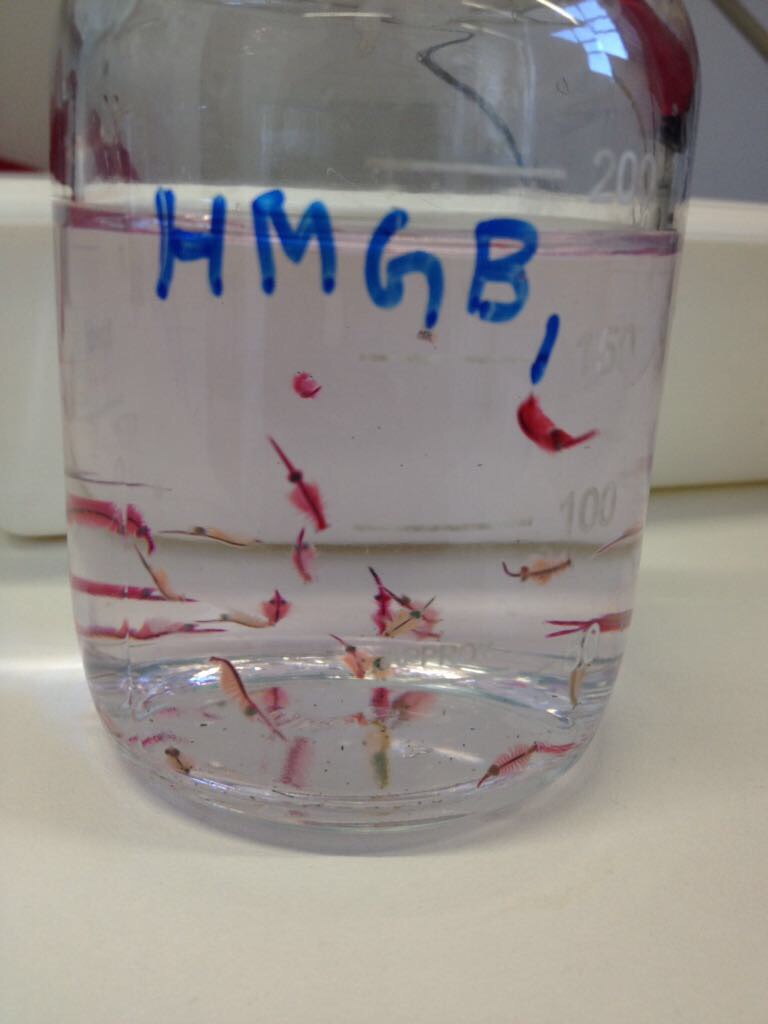


Figure S4 **Stained adult *Artemia* females post dsRNA micro-injection.** Total thirty numbers of females were injected with dsRNA for each *hsp70* and *gfp*. Injected females were observed for 2 h, and the animals which retained dye, remained healthy and could swim properly were employed for further experiments.


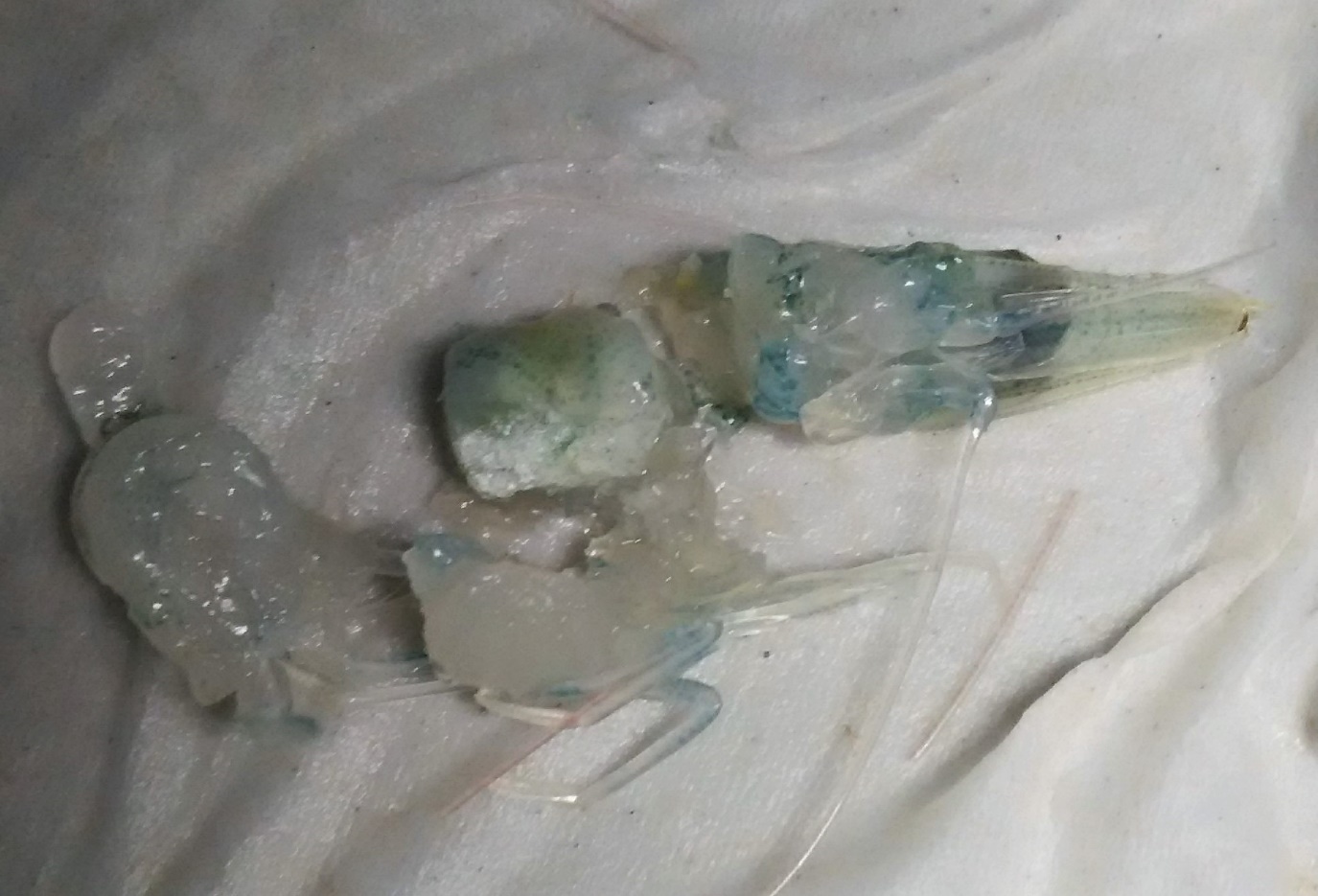


Figure S5 **Photograph of hepatopancreas from shrimp infected with AHPND (arrow)**. Gross signs include pale atrophied and white colour hepatopancreas.

Table S1 **Statistical analysis by two-way ANOVA of RNAi data**

| Source | df | F | Significance |
| --- | --- | --- | --- |
| Treatment | 1 | 30,303 | 0.00002 |
| *Artemia* type | 1 | 19,394 | 0.00027 |
| Treatment * *Artemia* type | 1 | 24,545 | 0.00008 |

*Artemia* type - positive control and phloroglucinol; treatment – dsRNA *gfp* (+ Hsp70) and dsRNA *hsp70* (− Hsp70)
